# Modeling Calcium Wave Based on Anomalous Subdiffusion of Calcium Sparks in Cardiac Myocytes

**DOI:** 10.1371/journal.pone.0057093

**Published:** 2013-03-06

**Authors:** Xi Chen, Jianhong Kang, Ceji Fu, Wenchang Tan

**Affiliations:** State Key Laboratory of Turbulence and Complex Systems and Department of Mechanics and Aerospace Engineering, College of Engineering, Peking University, Beijing, People’s Republic of China; University of Illinois at Chicago, United States of America

## Abstract

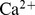
 sparks and 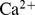 waves play important roles in calcium release and calcium propagation during the excitation-contraction (EC) coupling process in cardiac myocytes. Although the classical Fick’s law is widely used to model 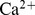 sparks and 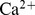 waves in cardiac myocytes, it fails to reasonably explain the full-width at half maximum(FWHM) paradox. However, the anomalous subdiffusion model successfully reproduces 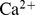 sparks of experimental results. In this paper, in the light of anomalous subdiffusion of 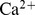 sparks, we develop a mathematical model of calcium wave in cardiac myocytes by using stochastic 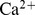 release of 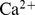 release units (CRUs). Our model successfully reproduces calcium waves with physiological parameters. The results reveal how 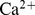 concentration waves propagate from an initial firing of one CRU at a corner or in the middle of considered region, answer how large in magnitude of an anomalous 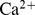 spark can induce a 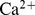 wave. With physiological 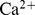 currents (2pA) through CRUs, it is shown that an initial firing of four adjacent CRUs can form a 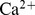 wave. Furthermore, the phenomenon of calcium waves collision is also investigated.

## Introduction

### Nomenclature


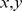
 spatial coordinates, 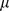
m


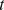
 time, ms


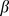
 fractional order of the spatial derivative.


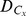
, 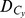 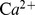 diffusion coefficients along 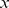-axis and 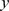-axis, 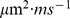



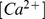
 free 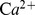 concentration, 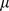
M


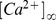
 resting 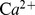 concentration, 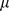
M


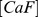
 Ca-bound fluo-3 concentration, 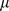
M


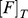
 total fluo-3 concentration, 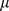
M


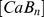
 Ca-bound endogenous buffer concentration, 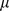
M


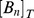
 total endogenous buffer concentration, 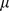
M


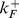
, 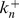 forward rate constants for dye and endogenous buffer reactions, 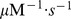



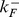
, 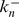 reverse rate constants for dye and endogenous buffer reactions, 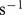



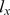
, 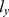 spatial separation of CRUs along 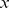-axis and 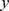-axis, 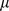
m


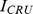
 current through the CRU, pA


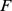
 Faraday’s constant, 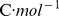



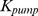
 SR pump Michaelis constant, 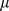
M


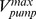
 maximum SR pump rate, 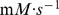



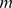
 SR pump Hill coefficient


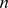
 CRU Hill coefficient


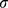
 molar flux of a clustered RyR channel, 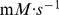



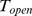
 open time of CRU, ms


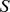
 stochastic switching function equaling either 0 or 1


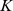
 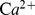 sensitivity parameter, 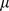
M


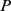
 probability of 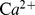 spark occurrence,/calcium release unit/ms


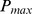
 maximum probability of 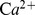 spark occurrence,/calcium release unit/ms


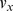
 wave velocity along 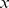-axis, 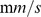



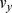
 wave velocity along 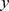-axis, 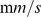


In the endoplasmic or sarcoplasmic reticulum(SR) of cardiac cells, there stores plenty of 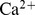, the concentration of which is 2–3 orders of magnitude greater than that in the cytosol. During the excitation-contraction(EC) coupling process, triggered by L-type 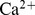 channels, 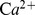 is released from SR through ryanodine receptors(RyRs) on the z-lines [1]–[4], where RyR is one kind of 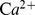 release units(CRUs). This event is called “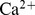 spark”. 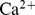-induced 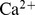 release(CICR) makes RyRs fire in succession such that 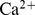 concentration rises [1], [5], the process of which is called calcium transient. Physiologically, calcium homeostasis is important for the contraction and relaxation of the heart muscle. However, in some pathological conditions, spontaneous propagating wave of 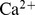 may occur, which is called “calcium wave”. The occurrence of calcium wave can affect the heart’s normal function, and may induce some disease, such as ventricular arrhythmias [6].

The model of 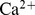 spark using Fick’s Law failed to reproduce the full-width at half maximum(FWHM) of experimental results for 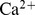 sparks. Simulated results for 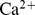 spark based on Fick’s Law presented a lower FWHM (∼1.0 *μm*), which was only half the width of experimental result (∼2.0 *μm*). Izu et al. [7] tried to increase the current through RyR to get larger FWHM, however, the spark amplitude also increased (∼10 times), which is far beyond experimental results and physiological conditions. In contrast, the results obtained with the anomalous subdiffusion model of 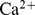 spark were found to be in close agreement with the experimental ones so that the “FWHM Paradox” was successfully explained [8]–[10]. Therefore, it is confirmed that diffusion of 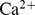 in cytoplasm obeys no longer Fick’s Law, but the anomalous subdiffusion.

A 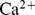 wave is formed from propagation of 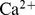 sparks. According to the results for 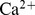 sparks, 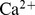 wave should also obey the anomalous subdiffusion. However, all previous work on 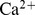 waves were based on Fick’s Law [11]–[14]. Anisotropic 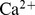 diffusion was studied by Girard et al. [11]. Keizer and Smith [12] investigated 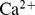 waves under stochastic firing of CRUs. Izu et al. [13] combined large CRU currents [7], stochastic firing of CRUs, asymmetric distribution of CRUs and anisotropic 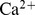 diffusion to investigate the propagation of 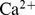 waves. Lu et al. [14] studied the effect of rogue RyRs on 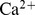 waves in ventricular myocytes with heart failure.

In this work, we develop a mathematical 2D model based on anomalous subdiffusion of 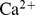 sparks. The anomalous subdiffusion model is used to study 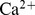 waves propagation from an initial firing of one CRU at a corner or in the middle of the considered region. We reproduce wave velocities of experimental results using a small current through CRUs which is close to the physiological conditions. The phenomenon of calcium waves collision is also investigated. With physiological 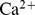 currents(2pA) through CRUs, an initial firing of four adjacent CRUs is shown to form a 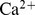 wave. Furthermore, study on how the system becomes unstable is also performed by changing the transverse distance of CRUs.

## Methods

### 0.1 Anomalous Diffusion Model for Calcium Waves

**Figure 1:**
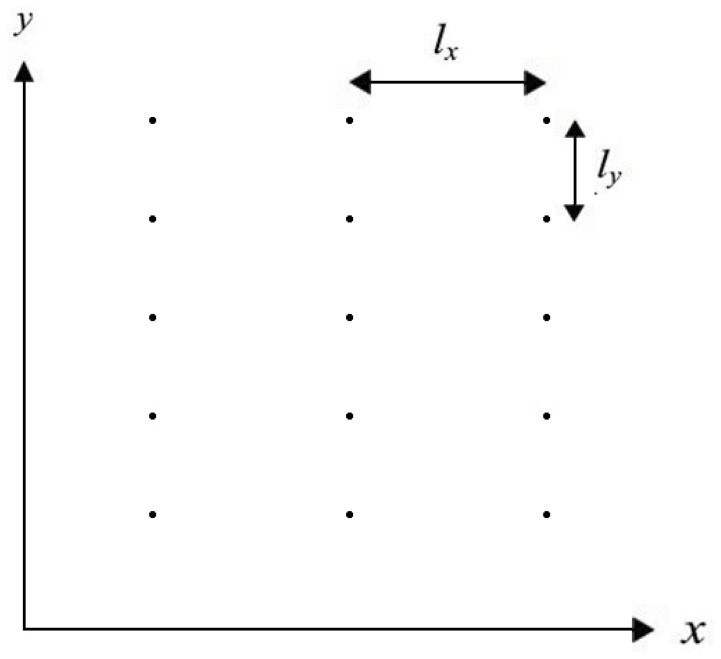
The 2D model of a cardiac myocyte. The black dots represent CRUs which distribute regularly spaced 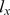 along 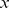-axis and 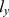 along 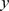-axis.



Figure 1 shows a 2-dimensional schematic of a cardiac myocyte(establishing line resources along 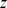-axis [13]) which contains plenty of CRUs. The regular intervals of CRUs are 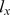 along 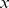-axis and 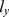 along 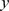-axis. The governing equation for 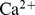 waves based on the anomalous subdiffusion model can be expressed as

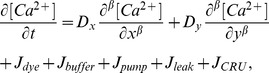
(1)where 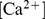 is the free 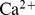 concentration; 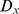 and 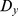 are diffusion coefficients for anisotropic diffusion with 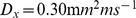 and 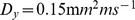 [15]; 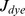 and 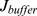 are fluxes due to 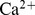 fluorescent indicator dye and endogenous stationary buffers; 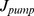 is pumping rate of SR 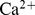-ATPase, and 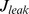 is a SR leak that is to balance 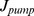; The expressions of 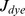, 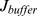, 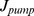, and 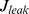 are
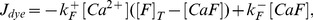
(2)

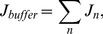
(3)

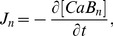
(4)

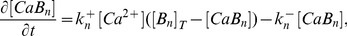
(5)

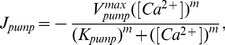
(6)

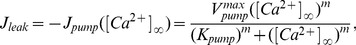
(7)where 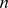 identifies each of buffer species. 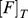 and 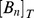 represent total concentration of the indicator and buffers, respectively. 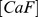 and 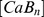 are concentration of the 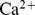-bound complexes. 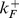, 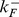, 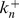 and 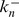 are reaction kinetics. 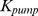 is the affinity constant for SR pumps, 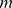 the Hill constant, and 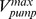 the maximum rate. SR leak is used to balance 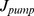 in resting state.


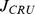
 is the flux of 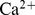 release from CRU, the expression of which is the same as that of Izu et al. [13],
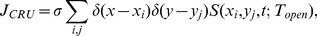
(8)where 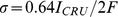 is a molar flux of a clustered RyR channel(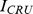 is current through the CRU and F is Faraday’s constant), and 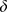 the Dirac delta function, S a stochastic function which controls the firing of the CRU, and 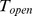 the firing time. Within a time interval 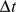, the probability that the CRU fires is 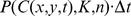, where 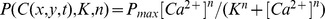 with 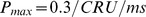 the maximum probability of 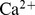 spark occurrence, 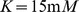 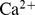 the sensitivity parameter and 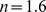 the Hill coefficient.

The anomalous space diffusion is model used in Eq.(1), where 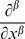 is the Riemann-Liouville operators which is defined as
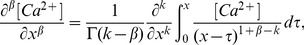
(9)

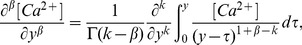
(10)where 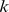 is an integer with 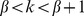 (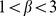). In Eq.(1), when 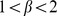, anomalous space superdiffusion occurs, while anomalous space subdiffusion occurs when 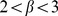. Particularly, our model reduces to Fick’s Law when 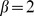. According to Li et al. [10], calcium sparks follow the anomalous space subdiffusion of 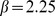, so we only consider the space subdiffusion in the following sections.

### 0.2 Numerical Methods

Our simulation is performed on a rectangular region with size of 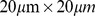, which is meshed with a uniform grid size of 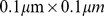. The time-step size is 0.005ms.

For the fractional differential term, we used the right-shifted Gr
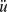
nwald formula to make a finite difference approximation [16].
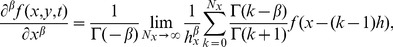
(11)

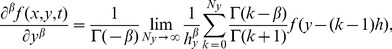
(12)where 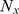 and 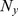 are positive integers, and 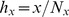, 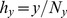. 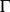 denotes the gamma function. The shifted Gr
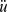
nwald approximation for fractional order derivative has been shown to be unconditionally stable [16].

Considering simple impermeability of the cell boundary to the diffusing ions, reflecting boundary conditions 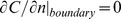 are taken on all edges [14]. The scale of our computation time is 200–500ms so that a CRU would not reopen after firing and closing.

Standard values of parameters used in the current study are listed in Table 1 and Table 2. 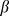, 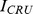 and 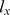 are changeable parameters whose effects on the results will be investigated.

**Table 1:** Standard parameter values.

Parameter	Value
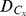	0.30
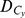	0.15
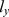	0.8
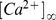	0.1
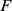	96500
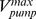	208
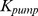	0.184
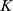	15
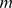	3.9
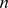	1.6
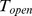	10
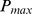	0.3

**Table 2:** Standard parameter values for dye and endogenous buffers.

buffers	*k^+^*	*k^­^*	[*B*]*_T_*
dye	80	90	50
Calmodulin	100	38	24
Troponin	39	20	70
SR	115	100	47
SL	115	1000	1124

## Results and Discussion

### Modeling a 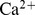 wave from a Single 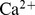 Spark


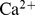
 waves have been shown to be initiated and sustained by 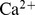 sparks [17]. Under pathological conditions, 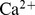 sparks fire spontaneously and stochastically, so whether a single spark can trigger a 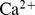 wave is important to the stability of cardiac myocytes [4]. Simulations based on Fick’s Law reveal that large currents through CRUs and high calcium concentrations are needed to trigger a 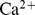 wave [13]. In this work, based on the anomalous subdiffusion model, we find the current which can trigger a normal 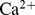 wave initiating from a single 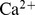 spark at the corner of considered region.

According to Li et al. [10], calcium sparks follow the anomalous space subdiffusion of 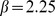, so this subdiffusion order is also taken in our simulation. In our model, initial source is a 10ms opening of one CRU, the longitudinal intervals are 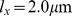. When we take 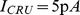, the longitudinal wave velocity (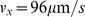 initiating from the corner) is in good agreement with the experimental result(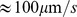 [17]).

**Figure 2:**
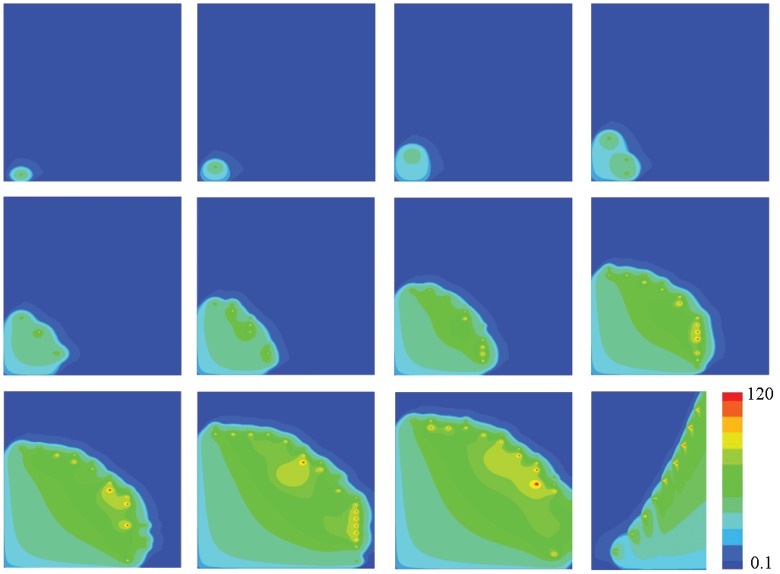
Snapshots of 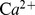 waves initiating from a 10ms opening of the CRU at the point (2,0.8). Snapshots are taken at 10, 30, 50, 70, 90, 110, 130, 150, 170, 190 and 200ms (left to right, top to bottom). Image 12 is the longitudinal linescan images along 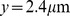. The value of parameters are 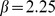, 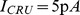 and 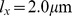. The concentration is from 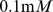 to 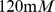.

Figure 2 shows 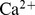 waves propagating on a discrete rectangle lattice initiating from a 10ms opening of the CRU at point (2,0.8). The snapshots are the 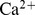 concentration distribution at 10, 30, 50, 70, 90, 110, 130, 150, 170, 190 and 200ms (left to right, top to bottom). From image to image, we can see CRUs fire stochastically by turns while 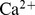 concentration wave propagates to the points of CRUs. At the beginning, CRUs fire one by one, and the amplitude(maximum of 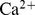 concentration in a region) is not very large; but with the increase of time, some of CRUs fire simultaneously in a short time so that 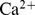 sparks influence each other, and the amplitudes of 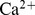 sparks become larger and larger. For example, the CRU at (2,1.6) fires at 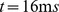, the CRU at (2,2.4) fires at 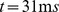; at 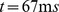, the CRUs at (4,0.8) and (4,2.4) fires simultaneously, in a short time interval, at 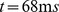 the CRU at (2,4.8) fires. In image 10, sparks occur at (18,4.0), (18,4.8), (18,5.6), (18,6.4), (18,7.2), (18,8.0), (18,8.8) and (18,9.6) in rapid succession, which may trigger a calcium transient. Image 11 shows that the boundary limits the propagation of 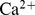 wave, but in an actual cardiac myocyte with size 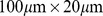, calcium transient will be observed. In addition, though the space intervals of CRUs along y-axis are more compact than along x-axis, transverse wave velocity 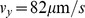 is smaller than that along x-axis 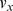 (because the diffusion coefficient along 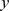-axis 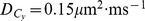 is smaller than that along 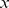-axis 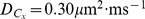).

Our numerical results show that a single 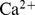 spark can trigger a normal 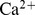 wave under the pathological condition of 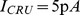 which is consistent with the experimental results(longitudinal wave velocity 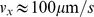). Physiological current through CRUs is about 2pA. However, the current may be increased (but not so large as 20pA [7], [13]) by external or internal factors, such as some disease or some electroneurographic signals. Our model present that a spontaneous 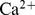 spark can form a 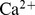 wave. The physical reason is, subdiffusion of 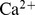 is slower than that for Fick’s diffusion. When an event of 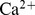 spark occurs, high value of 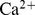 concentration may stay in a larger region around the firing CRU (for one 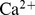 spark, FWHM is 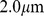 for subdiffusion and 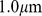 for Fick’s diffusion ), the firing probability of adjacent CRUs becomes higher. Then the fire-diffuse-fire process can be initiated and sustained, a 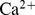 wave can propagate. So a smaller current and fewer sparks are needed to form a 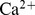 wave with our model than that using Fick’ Law.

Because of the large FWHM for one spark due to anomalous subdiffusion, one firing CRU will trigger a 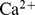 wave. Then we prohibit the event of another spontaneous spark so that it will not affect the initial 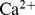 wave. In other words, when local 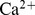 concentration is larger than resting 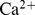 concentration, 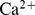 sparks may occur. So the “wall” of high 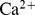 concentration spreads from the left corner to the top of cardiac myocyte. Image 12 shows the sequence of CRU firing along 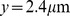
(the longitudinal linescan). The horizontal axis denotes time 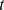
(from left to right,200ms), and the vertical axis denotes spatial coordinate 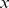
(20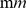). Except for initial two sparks, CRUs fire at nearly regular intervals, and the “wall” of high 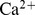 concentration is nearly a straight line. Here, from the initial spark to the second spark, it takes more time than those of the subsequent sparks, which is different from the results by Izu et al. [13](in their simulation, the transverse linescan is adopted, but the qualitative profile must be the same). It is because the subsequent sparks are triggered by two or more adjacent sparks, and the longitudinal wave velocity approaches to a constant value, but it takes more time to trigger the next CRUs from the initial signal spark.

### Effect of the Anomalous Subdiffusion Order

The anomalous diffusion order 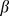, which determines the diffusion mode of 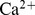 waves, was shown to affect the wave velocity considerably in last subsection(comparing with Fick’s Law). When 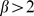, a large value of 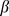 means a wild spread of initial concentration, but with the increase of time, the remanent concentration at initial point will be smaller due to the wild spread of calcium concentration. So the anomalous diffusion order 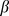 affects not only the wave velocity, but also the amplitude of each CRU, and further the average amplitude of a 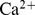 wave. Here, 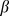 is taken to be 2.00, 2.05, 2.15 and 2.25 in order to figure out whether the amplitudes of 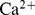 waves will change obviously with the variation of wave velocities. The initial condition is still a 10ms opening of the CRU at the point (2,0.8).

Table 3 presents the effect of anomalous fractional order 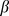 on the longitudinal wave velocity 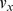 and the average amplitude, respectively. Comparing with the results based on Fick’s law, velocities of 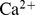 waves increase considerably when anomalous subdiffusion order 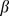 becomes bigger. For 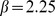, the wave velocity(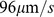) is almost twice as big as that based on Fick’s law(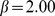, 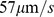). It is because FWHM along 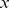-axis for 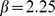 (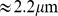) is almost twice as large as that for 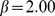
(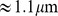). Here, in 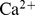 waves, FWHM for one CRU is affected by adjacent sparks, so it is a little bigger than FWHM of a single 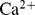 spark(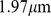 [10]). In contrast to wave velocity, the variation of amplitudes is not very considerable. For 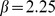, amplitude is 81% as that for 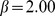. The physical reason is that although for 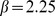, FWHM along 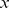-axis is twice as big as that for 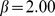, the full duration at half maximum(FDHM) along 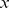-axis for one spark still has a obvious decrease. So when the total release of 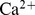 concentration is almost the same, under the expansion of spatial affection and the decrease of temporal continuity, amplitude of 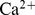 waves does not decreases obviously. In addition, wave velocities and amplitudes do not vary linearly with 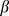. When 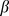 is larger, the effect of subdiffusion on 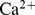 waves is greater. It is because when the variation of 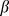 is small, the other parameters, such as the speed of diffusion 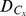 and the release strength of sparks 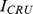, play important roles in 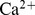 waves. When 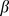 becomes bigger, replacing the primary position of the diffusion speed and release strength, diffusion mode affects 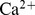 waves significantly(wave velocities).

**Table 3:** The effect of anomalous fractional order *β* to longitudinal wave velocity 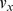 and amplitude.

*β*	2.00	2.05	2.15	2.25
ν*_x_*	57	62	75	96
amplitude	135	128	118	110

### Effect of Initial Location

Propagation of a 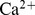 wave from a corner of the cardiac myocyte has been studied. It is found that the reflecting boundaries increase the amplitude of the initial spark, then further promote the propagation of the 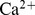 wave. In order to figure out the boundaries determine the propagation of the 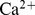 wave or just affect the wave velocity, we change the location of the initial 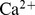 spark and study how the reflecting boundaries affect 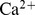 waves. In general, the process in which more than two sparks firing together, then several 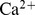 waves propagating, meeting and dissipating is very common in cardiac myocytes. This event is called 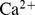 waves collision, and it was observed in experiments [17]. We will discuss in the following the interaction of several 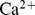 waves.

**Figure 3:**
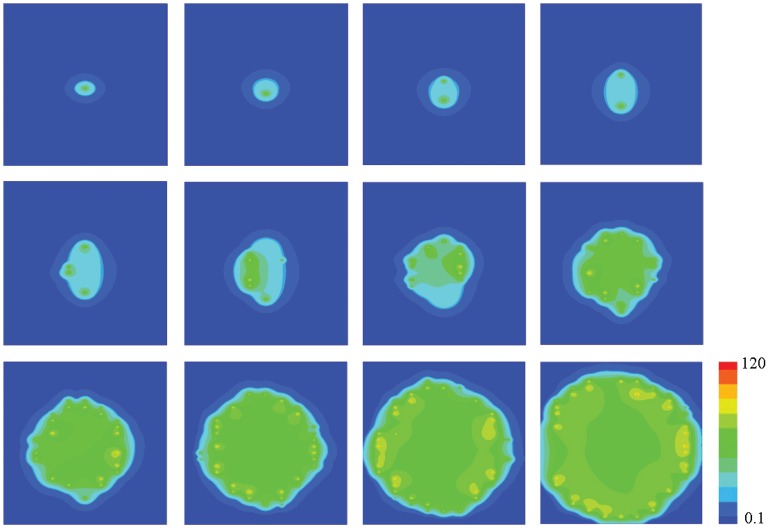
Snapshots of 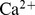 waves initiating from a 10ms opening of the CRU at the point (10,9.6). Snapshots are taken at 10, 20, 30, 40, 50, 60, 70, 80, 90, 100, 110 and 120ms (left to right, top to bottom). The value of parameters are 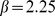, 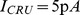 and 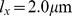. The concentration is from 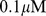 to 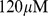.

As shown in Fig. 3, it takes only 120ms for a 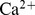 wave initiating from a 10ms opening of the CRU at a middle point (10,9.6) to propagates to the left and the bottom boundaries. Triggering from the middle of the region, the “walking distance” of a 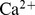 wave becomes shorter, and it will spread more quickly to the boundary. Due to the shorter “walking distance”, less sparks will occur simultaneously, and it will not make the 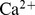 wave develop sufficiently; but trigger from the corner, while the 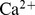 wave spreads wildly, large amount of sparks will fire together in a small region(Figure 2, right corner of Image 10). Comparing with the 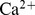 wave initiating from a corner without the effect of the boundaries, the initial concentration of the region will be smaller, then the probability of CRUs firing will be lower, so the events of 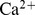 sparks are more stochastic and irregular. Affected by the absence of the reflecting boundaries and the shorter “walking distance”, longitudinal wave velocity 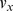 reduces to 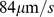, and 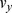 reduces to 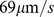. So 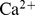 waves are easier to occur at the boundaries of cardiac myocytes, whcih can be compared with the experimental results [17], [18], [19]. Initiation of Free 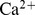 waves [18] and spontaneous 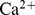 waves [19] is kinetically favored near the boundaries, and the waves initialing from the boundaries are also easier to propagate. In ref. [17], though the results are obtained by line-scan, initiation of the waves is always near the endpoints of the line, and the waves are always triggered near the boundaries of cardiac myocytes. However, the amplitude is smaller than that of the wave from the corner though the change is not obvious. It is because the initial condition is an opening of only one spark, the reflecting effects of the boundaries are not sufficiently obvious. So the reflecting boundaries can increase the propagating probability of 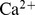 waves, though they are not the crucial factor for the propagation of 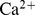 waves. In contrast, the anomalous subdiffusion mode of 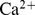 concentration is the decisive factor for whether the 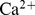 wave can be formed by a single 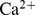 spark.

**Figure 4:**
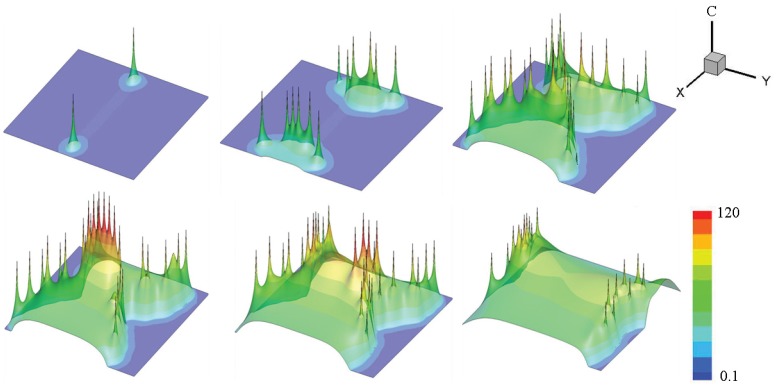
Snapshots of 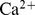 waves collision. Snapshots are taken at 10, 70, 110, 120, 130 and 150ms (left to right, top to bottom). The value of parameters are 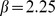, 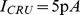 and 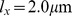. The concentration is from 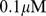 to 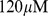.

Figure 4 presents the event of two 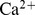 waves collision(3D images). The initial condition is 10ms opening of CRUs at points (2,9.6) and (18,9.6), and they will form two 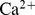 waves. Two 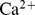 waves will meet at the middle as shown in Image 4, at 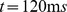. Several sparks fire at the same time, and local 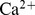 concentration reaches a peak value. With increasing time, 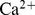 concentration will return to a lower value under the effect of buffers and pump, and no sparks will occur because of the CRUs’ “refractory period”. The 2D linescan image shows the process of two 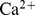 waves collision and vanishment(Fig. 8 in [17]), but it cannot show how the propagating direction changes. When 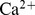 concentration reaches a peak value in the middle, CRUs along 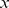-axis(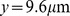) are closed, but CRUs along 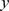-axis(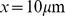) have never been opened before. Therefore, 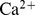 waves can propagate along the line of 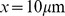. Finally, all CRUs are closed and will not reopen.

### Modeling 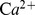 Waves Under a Physiological Current

To reproduce the feature of calcium waves found in experiments(primary result is wave velocity), a large current through CRU has been used in the former subsection. However, physiological value of 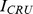 is about 2pA. So in the following discussion, 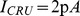 is adopted to study how many adjacent normal 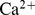 sparks can trigger a 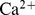 wave and find out the longitudinal interval of CRUs which could make a single 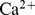 spark trigger a normal 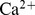 wave. Because of the small value of 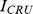, wave velocity and amplitude will be smaller. In order to make 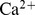 waves spread all over the region, the computation time is prolonged to 500ms. To diminish the effect of the reflecting boundaries, the initial location is chosen at the middle of the region.

**Figure 5:**
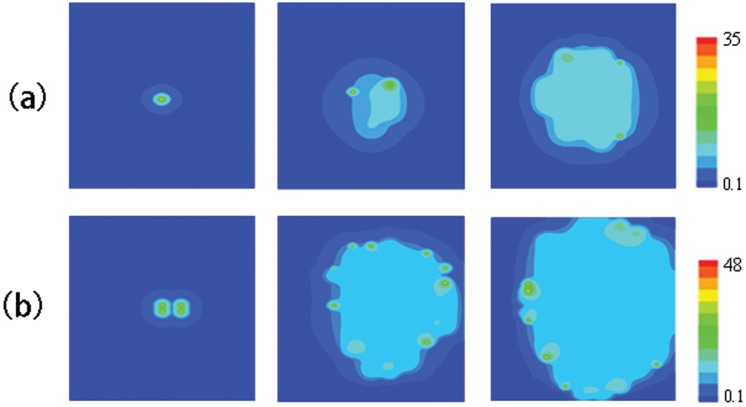
Illustration of. 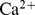 waves induced by physiological 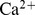 sparks. (a)Snapshots of 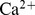 waves initiating from a 10ms opening of the CRU at the point (10,9.6), snapshots are taken at 10, 330 and 490ms. The value of parameters are 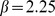, 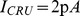 and 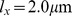. (b)Snapshots of 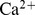 waves initiating from 10ms opening of the CRUs at the point (10,9.6), (12,9.6), (10,10.4) and(12,10.4), snapshots are taken at 10, 330 and 490ms. The value of parameters are 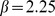, 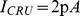 and 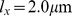.

Figure 5a shows whether a 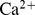 wave can be triggered by one spark at the middle of the region for 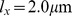. When 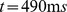, the wave only spreads through half the region, and the events of 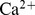 sparks are almost isolate. Under physiological conditions, even considering anomalous subdiffusion, high value of 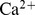 concentration may stay in a larger region around the firing CRU. For a small current, the amplitude of one spark is still small, the 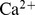 wave cannot propagate to the whole region, so the cardiac myocyte is stable when a normal spontaneous spark occurs.

The initial number of firing sparks is changed to study how many adjacent normal 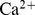 sparks can trigger a 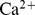 wave. The result is shown in Fig. 5b. It can be seen that four CRUs firing simultaneously at the middle will form a “weak” 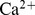 wave in the region. At 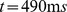, the wave reaches the top, bottom and right boundaries, and several sparks can be found at the same time. However, three adjacent normal 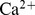 sparks can only form a local 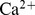 wave. So with the computation time of 500ms, for 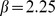, 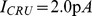 and 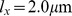, four adjacent CRUs firing together is the critical initial condition to trigger a 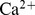 wave. However, wave velocity and amplitude here is very small(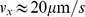), and the 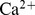 concentration of the whole region is much smaller than that in Figs. 2, 3, 4.

**Figure 6:**
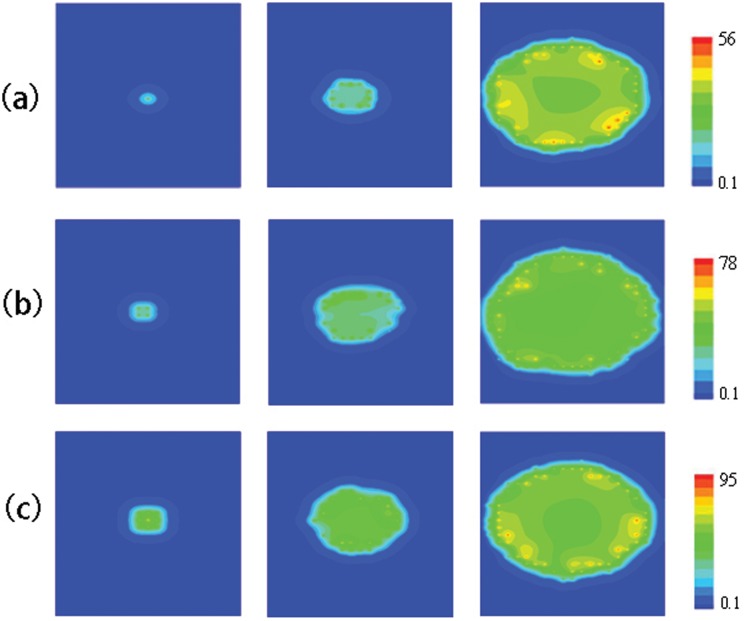
With smaller longitudinal intervals, the effect of initial 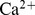 sparks numbers. (a)Snapshots of 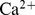 waves initiating from a 10ms opening of the CRU at the point (10,9.6), snapshots was taken at 10, 60 and 110ms. (b)Snapshots of 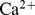 waves initiating from a 10ms opening of the CRUs at the point (9,9.6), (10,9.6), (9,10.4) and(10,10.4), snapshots are taken at 10, 50 and 90ms. (c)Snapshots of 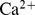 waves initiating from 10ms opening of the CRUs at the point (9,9.6), (10,9.6), (11,9.6), (9,10.4), (10,10.4), (11,10.4), (9,11.2), (10,11.2), (11,11.2),snapshots are taken at 10, 40 and 70ms. The value of parameters for a, b and c are 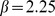, 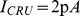 and 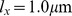.

In Figure 6a, the longitudinal interval is changed. For the case of 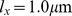, it takes only 110ms for the wave to reach the left boundary. With the simultaneous firing of several CRUs, an obvious 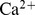 concentration wave is observed. Although the amplitude is smaller, longitudinal wave velocity(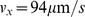) is comparative with the case of 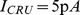, 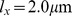. The physical reason for such a significant change which happens by changing 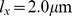 to 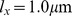 is that FWHM for 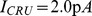 is about 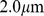, and if the interval between two CRUs reduces to 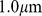, the half maximum value of a spark can “reach” adjacent CRUs easily. In addition, less interval makes more CRUs fire together, and the wave will be easier to propagate.

Our results have revealed that two factors(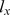 and number of firing CRUs) can both make a 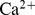 wave propagate. But which the effect is more significant? Figure 6 shows when 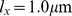, three 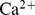 waves trigger from one, four, and nine initial adjacent 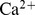 sparks, respectively. From 6a to 6c, both longitudinal wave velocity and amplitude become larger (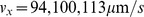, amplitudes are 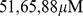), but the difference is not obvious as that between Figs. 5a and 6a. So the longitudinal interval of CRUs affects 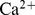 waves more significantly than the number of firing CRUs. If the longitudinal interval of CRUs becomes smaller due to some reasons, such as cardiac myocytes deformation, 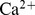 waves will easily occur, then cardiac myocytes will be unstable.

### Conclusion

In this work, we present a mathematical model based on anomalous subdiffusion of 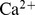 concentration in the process of 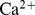 wave triggered by 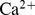 sparks. 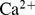 waves propagating from an initial firing of one single CRU at a corner or in the middle of a 2D rectangular region is numerically simulated. Our results can reproduce wave velocities of experimental results using a small current. We show that 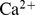 waves can be triggered by one single 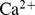 spark under a small CRU current(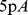). When anomalous subdiffusion order 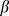 becomes bigger, velocities of 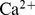 waves increase obviously, but the variation of amplitude is not very considerable. The phenomenon of calcium waves collision is also simulated. Under physiological 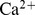 currents(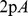) through CRUs, an initial firing of four adjacent CRUs is shown to form a 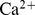 wave. When 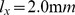, an isolated spark cannot trigger a 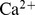 wave, so the system is stable under physiological condition. Then the longitudinal interval of CRUs is changed to study how the system becomes unstable and how an obvious 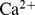 wave is formed. Our work is based on a more realistic diffusion model of 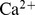 sparks with the parameters close to physiological values. The simulation results may be useful in further studies about 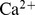 waves.
